# Cellular Response to Doping of High Porosity Foamed Alumina with Ca, P, Mg, and Si

**DOI:** 10.3390/ma8031074

**Published:** 2015-03-13

**Authors:** Edwin Soh, Elizabeth Kolos, Andrew J. Ruys

**Affiliations:** Biomedical Engineering, School of AMME J07, University of Sydney, Sydney, NSW 2006, Australia; E-Mails: edwin-soh@artc.a-star.edu.sg (E.S.); andrew.ruys@sydney.edu.au (A.J.R.)

**Keywords:** foaming, porous alumina, doping, cellular response

## Abstract

Foamed alumina was previously synthesised by direct foaming of sulphate salt blends varying ammonium mole fraction (AMF), foaming heating rate and sintering temperature. The optimal product was produced with 0.33AMF, foaming at 100 °C/h and sintering at 1600 °C. This product attained high porosity of 94.39%, large average pore size of 300 µm and the highest compressive strength of 384 kPa. To improve bioactivity, doping of porous alumina by soaking in dilute or saturated solutions of Ca, P, Mg, CaP or CaP + Mg was done. Saturated solutions of Ca, P, Mg, CaP and CaP + Mg were made with excess salt in distilled water and decanted. Dilute solutions were made by diluting the 100% solution to 10% concentration. Doping with Si was done using the sol gel method at 100% concentration only. Cell culture was carried out with MG63 osteosarcoma cells. Cellular response to the Si and P doped samples was positive with high cell populations and cell layer formation. The impact of doping with phosphate produced a result not previously reported. The cellular response showed that both Si and P doping improved the biocompatibility of the foamed alumina.

## 1. Introduction

Porous alumina can be used as a porous ceramic biomedical implant. Alumina was the first commercially significant bioceramic. It has been used in biomedical applications that require hardness, low friction and chemical stability, for example, dental implants and acetabular cup replacement in total hip prostheses. Other ceramics such as calcium phosphate do not have sufficient compressive strength in the porous form. 

The success of a porous implant depends on its ability to provide a functional balance between mechanical strength, pore size, interconnectivity of the porous structure and properties of osteoconductivity [1,2]. Mechanical strength is known to be reduced by porosity. 

The authors have previously published a method to produce porous alumina by foaming [3,4]. Porous alumina could be synthesised using *in situ* evolution of gases from calcining blends of ammonium sulphate and aluminium sulphate with varying ammonium mole fraction (AMF). High levels of porosity 94%–96% were achieved with acceptable mechanical strength of 380 kPa which is comparable to other biomaterials of similar porosity levels.

To make the bioinert alumina material bioactive and thus potentially more suitable for the biomedical application, doping of porous alumina has been considered. For this study, doping with calcium (Ca), phosphate (P), Silica (Si) and Magnesium (Mg) and combinations at low and high concentrations was trialled.

Calcium and phosphate coatings are known to be bioactive and therefore doping with Ca and P was trialled. Incorporation of magnesium ion in an alumina implant has previously been shown to improve bone cell adhesion [5]. Silica doping of hydroxyapatite and its improved cellular response has been vastly reported [6,7,8,9,10]. Silica doping of alumina tubes with small amounts of Si has previously been shown to improve tissue ingrowth, differentiation and osteogenesis *in vivo* [11]. 

The combination of the foamed alumina containing high porosity, pore size, pore interconnectivity and strength when doped to improve bioactivity could uniquely combine the properties required for biomedical applications.

The key focus of this study is to take an optimal product from the foaming method used to produce a high porosity (94%) alumina, average pore size of 300 µm, with a degree of pore interconnectivity and high compressive strength, dope it with Ca, P, Si, Mg and test the cellular response compared to the control foamed alumina. 

## 2. Materials and Methods

### 2.1. Synthesis of Porous Alumina

**Figure 1:**
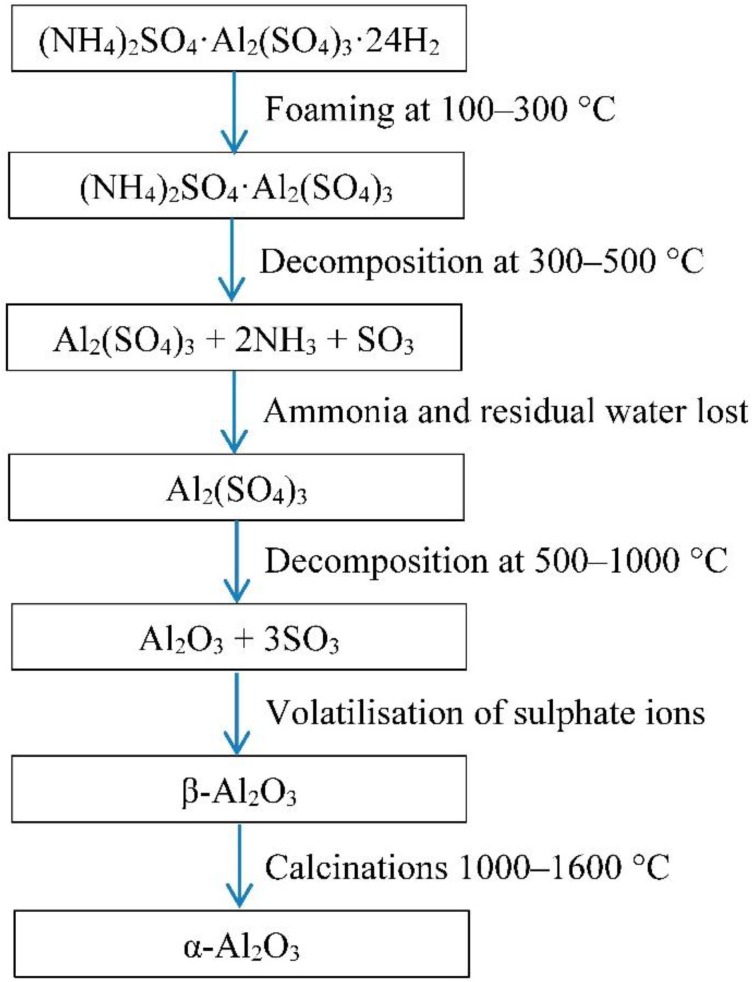
Chemical synthesis of aluminium sulphate and ammonium sulphate solution.

Porous alumina was synthesised using the chemical breakdown of ammonium sulphate and aluminium sulphate salt solutions [3,4]. This method is known as the direct foaming of sulphate salt blends. The sulphate mixture undergoes a complex heating cycle in which it is first volatised, calcined and finally sintered, producing a strong porous structure. See Figure 1.

Aluminium sulphate and ammonium sulphate were homogeneously mixed in an air tight bag before distilled water was added. The sulphate salt blends, known as alumina precursor, were dissolved in water and preheated before foaming. Heating of the solution for foaming reaches boiling temperature, foaming occurs with the evaporation of excess water content. With increasing temperature, the ammonium starts to decompose causing ammonia and residual water to be lost. In the final decomposition state, sulphate ions were volatised and porous alumina was obtained. The green body porous alumina was then removed from the crucible and sintered in a high temperature furnace, where the transformation β-alumina to α-alumina occurs. 

Previously, ammonium sulphate mole fraction in the aluminium/ammonium sulphate blend, referred to as the ammonium mole fraction (AMF), foaming temperature and the sintering temperature were varied [4]. The optimal product was produced with 0.33AMF, foaming at 100 °C/h and sintering at 1600 °C. This product attained high porosity of 94.39%, large average pore size of 300 µm and the highest strength of 384 kPa. 

To attain the optimal product 0.33AMF, the compositions of salt used were 13.21 g of ammonium sulphate, 133.29 g of aluminium sulphate and 36 g of distilled water.

### 2.2. Doping of Porous Alumina

#### 2.2.1. Ionic Doping with Calcium, Phosphate and Magnesium

Porous alumina blocks were bulk doped with calcium (Ca), phosphate (P) and magnesium (Mg) at dilute (10%) and saturated (100%) concentrations. They were soaked in the solution for 24 h and dried to 900 °C with a step increment of 100 °C/h.

The main stock (100% concentration) of calcium, phosphate and magnesium solutions were made by adding excess salt to distilled water at room temperature and allowed to settle before decanting the salt solution into a glass container. Dilute solutions (10% concentration) were made by diluting the stock solution with suitable parts of distilled water. The following salts were used for making the stock solution: Calcium nitrate tetrahydrate, Ca(NO_3_)_2_; Ammonium dihydrogen phosphate; (NH_4_)_2_HPO_4_ and Magnesium nitrate, Mg(NO_3_)_2_·H_2_O.

Table 1 shows the weight% of salt in the solution for Ca, P and Mg. Equal parts of the respective concentrated solutions were used in mixture of CaP and CaPMg solution.

**Table 1:** The weight % of salt in the solution for dilute (10% concentration) and saturated (100% concentration).

Dilute	Wt%	Saturated	Wt%
Ca	0.059	Ca	0.59
P	0.028	P	0.28
Mg	0.056	Mg	0.56

#### 2.2.2. Silica Doping 

Silica used for doping of porous alumina was made via the sol gel method. Porous alumina specimens are soaked for 24 h in the silicon alkoxide precursor containing tetraethyl orthosilicate Si(OCH_2_CH_3_)_4_, commonly known as TEOS, with ethanol at room temperature. A catalyst solution containing aqueous ammonia, ammonium fluoride and distilled water was then slowly added to the TEOS solution and left to soak for a further 24 h before drying in an air furnace at 900 °C. Table 2 shows the composition of solution and quantity used for silica doping. 100% concentration only was done for silica doping. 

**Table 2:** The composition of solution and quantity used for silica doping.

Silica Solution	TEOS	50 mL
	Ethanol	40 mL
**Catalyst Solution**	Ethanol	3 5mL
	Water	70 mL
	Ammonia (30%)	0.275 mL
	Ammonium Fluoride (0.5 M)	1.21 mL

### 2.3. Cell Culturing

#### 2.3.1. MG 63 Cell Line

MG-63 cells are derived from osteosarcomas, malignant bone tumours consisting of cells with abnormal cellular functions, and are commonly used for osteoblastic models as the cell synthesises osteoid and exhibits increase alkaline phosphatase and osteocalcin hence provides a good cellular model for testing bone implant materials. 

#### 2.3.2. Cell Culture

All alumina specimens were sterilized in 70% ethanol for 30 min and exposed to ultraviolet (UV) light prior to being introduced into 96 well culture plates. Alumina specimens of 4 mm diameter were attached to the culture plates with Vaseline^®^. MG3 cells were seeded onto the specimens along with Dulbecco’s Modified Eagle Medium (DMEM) containing calcium (Ca^2+^), supplemented with 10% foetal bovine serum (FBS) at a density of 15,000 cells/well. The plated specimens were then incubated at 37 °C in a humidified atmosphere of 95% air and 5% carbon dioxide for a period of 3 days. 

#### 2.3.3. Cell Viability

Cell viability of the synthesised porous alumina was assessed by the number of healthy cells in the seed specimens. The MG63 cells were rinsed with Phosphate Buffered Saline solution (PBS) and trypsinised for 8 min at 37 °C. Once cells were detached from the surface, the solution was neutralised with 50 µL of FBS followed by staining using 100 µL of Trypan Blue. The cell suspension was flushed several times for even cell distribution before pipetting 10 µL into a haemocytometer for counting under a light microscope. Cell viability was done in triplicate. 

#### 2.3.4. Cell Morphology

Cell morphology indicates the health of the cell and how well it responds to the chemistry and topography of the underlying substrate. Cultured cells were rinsed with pre-warmed PBS solution and fixed onto the porous specimens using 2% glutaraldehyde at room temperature for 1 h followed by dehydrating in a series of graded ethanol solutions (50%, 70%, 80%, 90%, 95% and 100%) for 10 min. The fixated cells were then stained with osmium before being critical point dried with the Bal-Tec CPD 030 Critical Point Dryer (CPD). The prepared alumina specimens with fixated cells were then sputter coated with gold to a thickness of 10 nm before being observed in the scanning electron microscopy (SEM).

### 2.4. Characterisation

#### 2.4.1. SEM

The microstructure of synthesised porous alumina was observed with the Philips XL30 SEM (FEI, Eindhoven, The Netherlands). A high energy beam of electrons, with an average accelerating current of 15 kV interacts with the atoms on the specimen surface. Pore size and cell wall thickness were measured with inbuilt measurement software. To prevent the accumulation of electrostatic charge on the surface, specimens were sputter coated with gold before imaging. 

#### 2.4.2. EDS

The Philips XL30 with an EDAX-ZAF EDS system (FEI, Eindhoven, The Netherlands) operating at 10 kV was used to determine the elemental composition of samples.

## 3. Results

### 3.1. Doping

Observation by SEM and EDS was done for foamed porous alumina samples doped with Ca, P, Mg with dilute (10% concentration) or saturated (100% concentration) and alumina samples doped with Si concentration of 100%.

**Figure 2:**
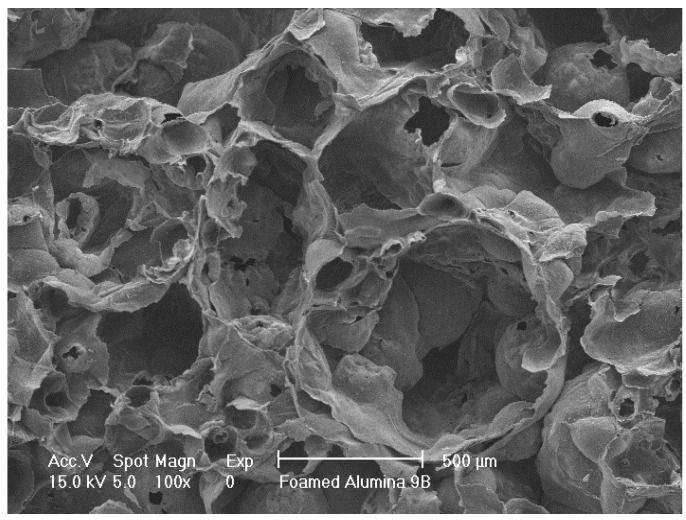
Scanning electron microscopy (SEM) of sintered porous alumina.

SEM of control foamed alumina without doping is shown in Figure 2. 

**Figure 3:**
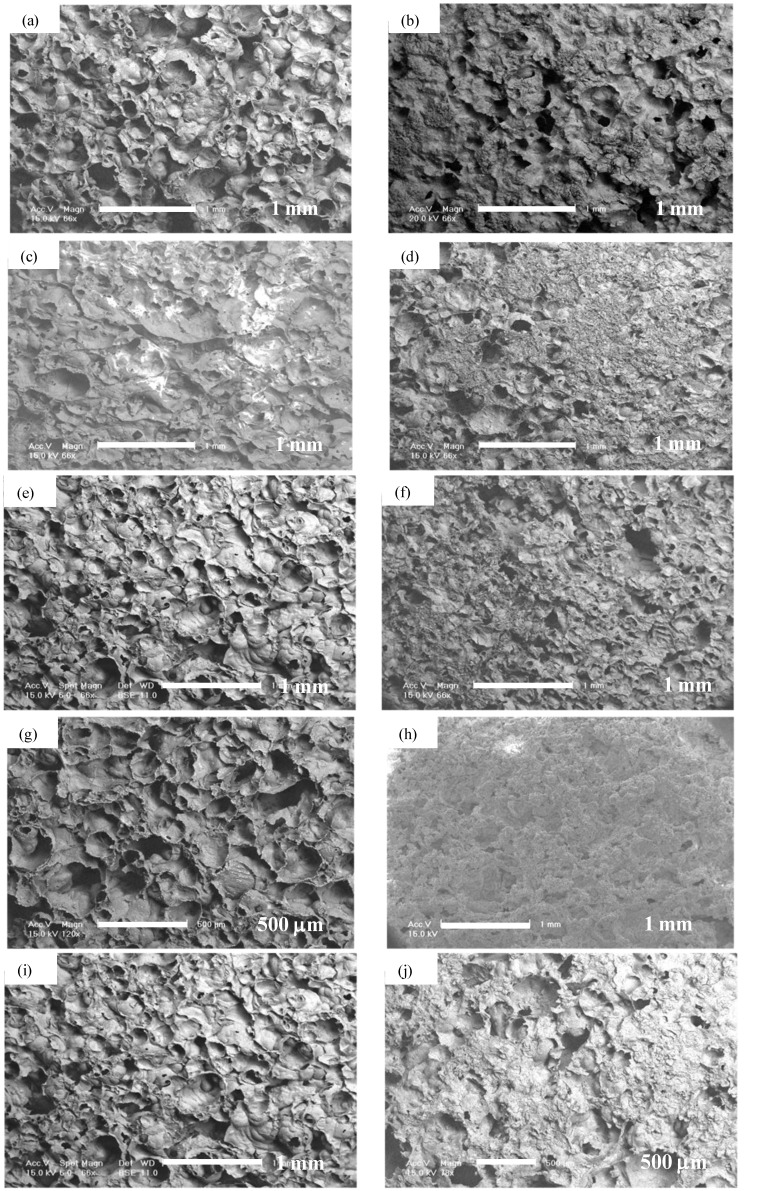
SEM of porous alumina with 10%Ca (**a**); 100%Ca (**b**); 10%P (**c**); 100%P (**d**); 10%Mg (**e**); 100%Mg (**f**); 10%CaP (**g**); 100%CaP (**h**); 10%Ca + P + Mg (**i**); 100%Ca + P + Mg (**j**).

**Figure 4:**
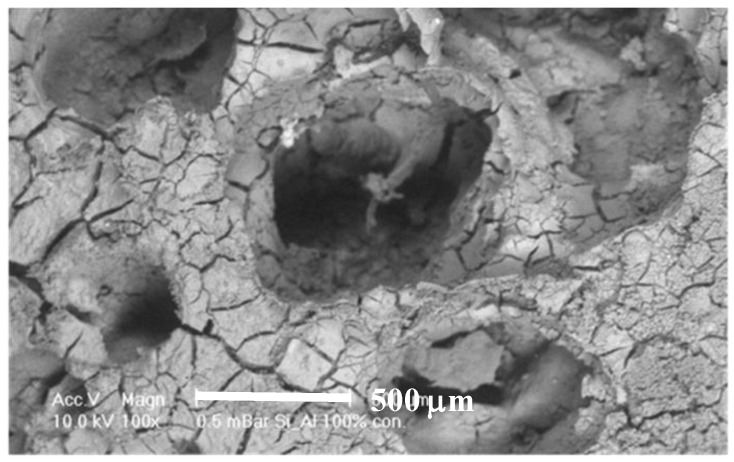
SEM of porous alumina with 100% Si.

Porous alumina doped with 100% and 10% solutions of calcium (Ca), phosphorous (P), magnesium (Mg), Ca + P and Ca + P + Mg were immersed in concentrated solution for 24 h before drying. Precipitates could be found distributed on surfaces treated with saturated concentrations of dopants. There were minimal precipitates visible on surfaces with dilute concentrations of dopants. Silica (Si) doped porous alumina showed homogeneous surface distribution. SEM micrographs of treated surfaces can be seen in Figure 3 and Figure 4. 

The surface compositions of the doped samples as tested by EDS are presented in Table 3. Numerical results from EDS are not definitive, however results do show that dopant was present.

**Table 3:** EDS composition results showing dopant present on alumina samples.

Dopants	Concentrate %	Element wt%				
		Al	Ca	P	Mg	Si
Ca	10	77.78	22.22	–	–	–
	100	10.20	89.80	–	–	–
P	10	93.42	–	6.58	–	–
	100	60.69	–	39.31	–	–
Mg	10	89.63	–	–	10.37	–
	100	65.05	–	–	34.95	–
Ca/P	10	76.75	14.25	9.00	–	–
	100	15.80	60.59	23.61	–	–
Ca/P/Mg	10	89.90	5.84	2.61	1.62	–
	100	17.46	44.75	26.08	11.7	–
Si	100	79.06	–	–	–	20.94

Cellular response studies were conducted on these samples to quantify the viability of the doped surfaces. 

### 3.2. Cell Viability

**Figure 5:**
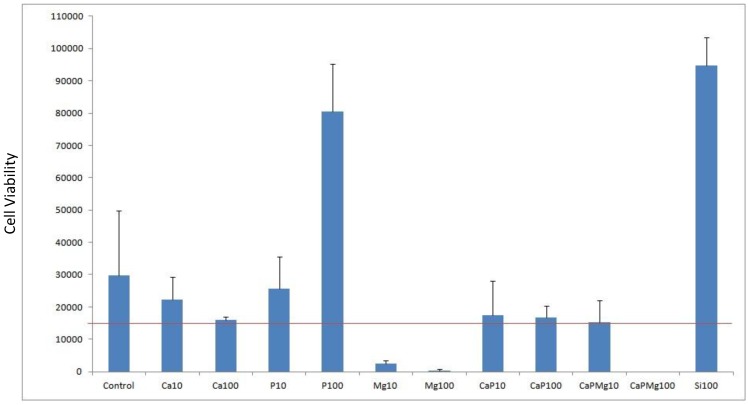
Plot of cell viability for doping alumina with Ca, P, Mg, Si as well as mixtures used with a concentration of 10% and 100%. Porous alumina without doping used as control specimen. Red horizontal line represents the seeded density of cells count.

Cell viability was done by cell counts of MG63 seeded on to porous alumina as a control and with dopants Ca, P, Mg, Ca/P, Ca/P/Mg and Si to test biocompatibility. The result shown in Figure 5 shows the effect that dopant or dopant concentration has compared with the control alumina material. The red line represents the density of cells seeded at 15,000 cells per well. 

After the culturing period of 3 days, most dopant concentrations demonstrated positive cell counts above the seeded density. However samples containing Mg10, Mg100 and CaPMg100 demonstrated negative cell counts above the seeded density. These specimens containing magnesium content suffered a significant drop in cell counts below the seeded density. Increasing magnesium concentration from 10% to 100% saw a severe drop in cell counts from 2333 to 333 cells per well. When magnesium of 100% concentration was added to the concentrated calcium phosphate doping (CaPMg100), no living cells were found due to the high concentration of magnesium content making the environment unviable for the cells. 

Specimens doped with 100% phosphate (P100) and 100% silica (Si100) concentration achieved cell counts 4 to 5 times greater than the control specimens with counts reaching 80,500 and 94,833 cells respectively. 

Porous alumina doped with 10% (Ca10) and 100% (Ca100) calcium concentration showed decreases in cell count compared to the control specimen with 29777 cells per well. Ca10 however had a higher count than Ca100 with cell counts almost similar to the seeding density. 

The addition of 100% phosphate (CaP100) increased cell count slightly from 16000 to 16666 cells.

### 3.3. Cell Morphology

Porous alumina as control and doped with Ca, P, Mg, Ca/P, Ca/P/Mg and Si were seeded with MG63 cells and examined after 3 days of culture with SEM. Observations made on both P and S doped samples with high cell populations showed cell layer formation. 

**Figure 6:**
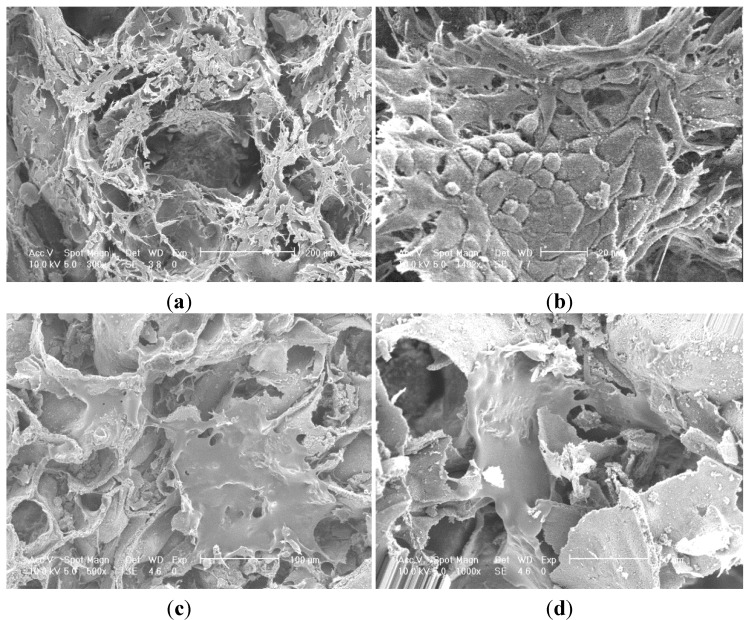
SEM micrographs cell layer formation (**a**) phosphate showing migration of cells into porous network; (**b**) phosphate showing morphology of confluent layer at high magnification with squamous appearance; (**c**) silica showing two cell monolayers spanning across pore openings; (**d**) silica showing two monolayer of cells migrating towards each other along the wall of the silica doped alumina.

SEM images of P100 and Si100 are presented in Figure 6.

Observations made on both P and Si doped samples with high cell populations showed cell layer formation. Figure 6a showed a confluent layer of cells formed on both internal and external surfaces of the P doped sample. Similar results were observed for the Si doped sample but with numerous small patches of cells with flattened morphology forming multiple small monolayers on the surfaces of the pore channel as seen in Figure 6c.

Cells had elongated morphology with spindle shaped cellular extensions with rough dorsal surface representing characteristics of active cells.

Cell adhesion was characterised by cell-to-surface adhesion and cell-to-cell where both cell-to-surface adhesion and cell-to-cell adhesion was observed. 

The Si doped sample in Figure 6c presents evidence that the fibrous cell layer has stretched across a pore opening and adhered throughout the pore cavity forming a suspended web like structure. There is visible filopodia attached to the microstructure surface.

P doped samples had cells attached in the pore cavities however there was some evidence of a monolayer. Cells have extended across boundaries and adhered to the surface by filopodia attachment.

**Figure 7:**
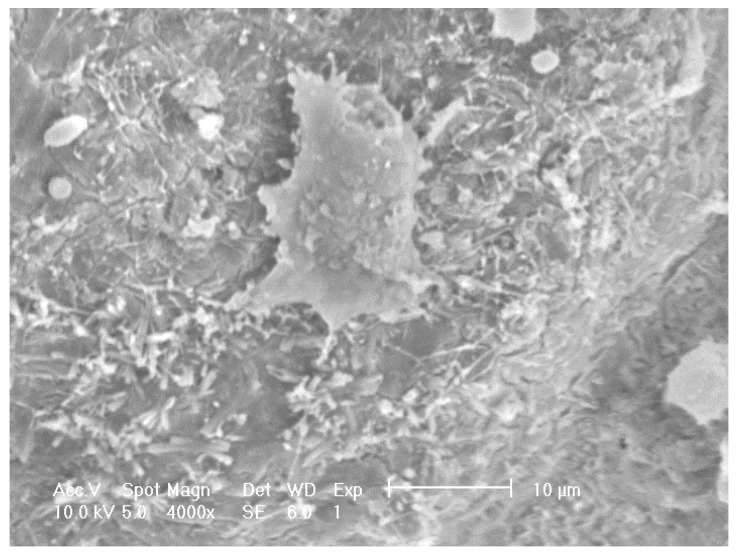
SEM images revealing the morphology of MG63 cells seeded on to porous alumina doped with Calcium.

SEM on other doped samples showed sparse distribution, for example Figure 7 shows foamed alumina doped with Ca with three cells on the inner surface of a pore cavity with occasional cell clusters. Adhered cells with extended filopodia were found on the Ca doped samples although they appeared to be partially attached to the substrate. Cells appeared to be more rounded and less healthy when compared to the cells seen on samples doped with P or S.

Some damage from the dehydration process during preparation for SEM was observed and caused some cell shrinkage. 

Viability results on cultured samples doped with Ca and CaP were found to have neutral cellular responses, while samples doped with Mg were negative. Samples doped with Si and P were found to be strongly positive. Good cell to cell and cell to surface adhesion with high population of cells, cell migration and cell confluence were found on both Si and P doped samples with Si doped samples producing a higher degree of cell confluence. 

## 4. Discussion

Doping of foamed porous alumina using saturated (100%) and dilute (10%) concentrated solutions of calcium (Ca), phosphorous (P), magnesium (Mg), Ca + P and Ca + P + Mg were done. The results indicated that doping of the foamed alumina could be done and that soaking in saturated solutions produced more dopant adhering to the surface. 

Precipitates were observed on the surface of the samples and thus surface modification of the porous alumina was achieved. Precipitates could be found homogenously distributed on surfaces treated in saturated solutions of dopants. There were minimal precipitates visible on surfaces treated in dilute solutions of dopants. Precipitates were in the size range of approximately 10 μm. Silica (Si) doped porous alumina showed homogeneous surface distribution. Si doping produced a visually thicker and denser layer. 

EDS results, although not numerically definitive, suggests dopant was present. The surface effect of doping the porous alumina is further discussed in relation to cellular response. 

To test the biocompatibility of the foamed alumina as a control and the impact of doping the foamed alumina, cell culture using MG63 cells was done. 

Cell viability results in Figure 5 showed positive growth rates of cultured cells on both Si and P doped samples. Cell morphology as seen in the SEM micrographs in Figure 6 also showed large cells population well distributed on both Si and P doped surfaces. Si doped surfaces did achieve a higher degree of cell confluence with interactions between adjacent cell monolayers after only 4 days of cell culture. There were partially foamed cell monolayers found on the P doped samples surrounded by large neighbouring cell clusters. Cells could be seen in the porous structure of the alumina specimen which suggests that the average pore size of 300 µm was sufficient for cell migration and proliferation to take place. 

The impact of silicon doping of hydroxyapatite and its improved cellular response has been vastly reported [6,7,8,9,10]. Calcium phosphate ceramics such as hydroxyapatite that are substituted, doped, coated or merely contain traces of Si in the form of silica, silicon or silicate have been shown to improve cellular response. It is thought the mechanism of this Si substitution promotes biological activity by the transformation of the material surface to a biologically equivalent apatite by increasing solubility of the material by generating a more electronegative surface which is mediated by serum proteins and osteoblast-like cells inducing dose-dependent stimulatory effects on cells and bone/cartilage tissue systems [10].

The impact of doping alumina tubes with small amounts of Si significantly improved tissue ingrowth, differentiation and osteogenesis *in vivo* has been reported [11]. Pabbruwe *et al.* suggest that the effect of Si is related to surface chemistry rather than microstructure. 

It is purported that a similar mechanism at the alumina’s surface with Si substitution into the alumina crystal structure as is suggested for Si-substituted HA could occur in the current study. 

The impact of phosphate doping of alumina has not been previously reported. Although phosphate is a key bone nutrient the degree of cell confluence achieved was unexpected. 

A possible explanation could be found by looking at biocompatible phosphate glasses. Phosphate glasses are biocompatible with a human skin fibroblast model depending on their solubility and testing procedure [12]. The degree of solubility from the phosphate glasses had an effect on *in vitro* behaviour. The glasses without TiO_2_ and therefore more soluble had more fibroblast cell adhesion. Narvarro reported that phosphate glasses with highly soluble phosphate induced a kind of extracellular protein secretion and synthesis of a matrix protein [12].

These results are in agreement with those reported by Salih *et al.* who found that human osteoblast cell lines cultured in the presence of extracts of a phosphate glass (with a chemical composition very close to Narvarro et al samples with higher solubility) resulted in an enhancement of bone cell proliferation and an upregulation of the expression of sialoprotein, osteonectin, and fibronectin [13].

Narvarro et al reports that the reasons that could explain these effects are not clear but must be related to the different chemical composition and dissolution behaviour of the glasses, which corroborates that solubility is a main point in the material-cell interactions [12]. Narvarro et al then concludes that although it is not clear which of the released ions can be responsible for the observed effects, it is suggested that calcium could play a significant role in protein secretion [13,14]. This current study would confirm that phosphate rather than calcium produced a better cellular response.

The doping of phosphate on the alumina likely led to the formation of aluminophosphate which is essentially an inorganic phosphate. Beck, G.R. has reported that the generation of inorganic phosphate during the process of osteogenic cell differentiation may act as a signalling molecule for mineralizing cells to respond to the changing extracellular environment by regulating protein function and gene expression [15]. This may provide another explanation for the positive cellular response of phosphate.

Doping alumina with Ca produced a poorer result than the control as seen in Figure 6 but did not reduce the cell density from the number seeded. Comparing the 10% (Ca10) and 100% (Ca100) showed that increasing the calcium concentration decreased cell count. This suggests that the higher concentration of calcium present in the culture medium led to the poisoning of cells. Figure 7 shows cells present and adhered to the surface but did not attain confluence as Si and P doped samples did. Therefore while intracellular Ca^2+^ transients have been implicated in most aspects of cell physiology, including gene transcription, cell cycle regulation and cell proliferation, it is known that the Ca^2+^ ion has been found to play an important role in cell death regulation by Ca^2+^ signalling [16]. 

Pabbruwe et al also found the addition of Ca to alumina promoted hypertrophic bone formation at the advancing tissue fronts and appeared to retard angiogenesis by limiting ongoing cellular migration *in vivo* [17]. Pabbruwe et al speculated that the presence of a secondary phase of calcium hexaluminate, probably having a solubility greater than that of alumina, possibly increased the level of extracellular Ca and, consequently, stimulated osteoclastic activity at the bone-ceramic interface [17]. 

Doping alumina with CaP produced a poorer result than the control as seen in Figure 6 but did not reduce the cell density from the number seeded. Comparing the 10% (CaP10) and 100% (CaP100) showed that increasing the calcium and phosphate concentration decreased the cell count slightly. This result could suggest that a calcium phosphate phase did not form on the alumina to produce a bioactive coating. But rather as with the Ca doping, the calcium ions present limited the cellular response by the same mechanism as referred to above.

The poor cellular response for magnesium doped samples was most likely caused by magnesium poisoning, suggesting that magnesium is only helpful when it is used as a trace element in conjunction with other bioactive ions combined. Other metallic ions manganese and chromium used for doping of alumina produced a positive cellular response *in vivo* [17]. However those results were not replicated in this study with magnesium. While magnesium is known to have a stimulatory effect on bone formation [5], the role that magnesium plays may involve trace quantities or not occur in isolation. Therefore magnesium was not suitable for doping of alumina in a biological application using the above method. 

## 5. Conclusions

Foamed alumina was previously synthesised by direct foaming of sulphate salt blends varying AMF, foaming heating rate and sintering temperature. The optimal product was produced with 0.33AMF, foaming at 100 °C/h and sintering at 1600 °C. This product attained high porosity of 94.39%, large average pore size of 300 µm and the highest strength of 384 kPa.

This study doped the foamed alumina with different concentrations (10% and 100%) of Ca, P, Mg, CaP and CaP + Mg and Si. Doping could be successfully done and results were presented with EDS and SEM.

Cellular response was tested with cell culture of MG63 osteosarcoma cells with a control alumina as well as the doped surfaces. Cellular response to the Si and P doped samples was positive with high cell populations and cell layer formation as seen with cell viability and cell morphology studies. 

In conclusion, the impact of doping with phosphate produced a result not previously reported and the cellular response showed that both Si and P doping improved the biocompatibility of the foamed alumina. 
